# Pour une communication basée sur la culture en santé (*health literacy*) des populations

**DOI:** 10.48327/mtsi.v2i3.2022.185

**Published:** 2022-09-28

**Authors:** Bernard SEYTRE

**Affiliations:** 1 bnscommunication, 7 rue Ledion, 75014 Paris, France

**Keywords:** Health literacy, Culture en santé, Afrique subsaharienne, Health literacy, Health culture, Sub-Saharan Africa

## Abstract

Le concept de « *health literacy* » est très utilisé dans les pays anglophones, où il sert de cadre théorique à des approches de l’éducation en santé, mais encore peu connu dans les pays francophones et, semble-t-il, ignoré en Afrique francophone. Nous proposons de l'appliquer à la communication en santé en le traduisant par « culture en santé », expression très large qui englobe les connaissances et les représentations, non seulement sur des questions de santé mais aussi sur les autorités sanitaires et les promoteurs des messages de santé publique. L'approche de culture en santé permet de fonder des stratégies, messages et outils de communication sur les représentations de la population ciblée, pour l'amener à adhérer aux changements de comportement promus par cette communication. Nous montrons quelques exemples de son application dans des programmes de santé publique en Afrique.

## Abstract (en)

The concept of “health literacy” has been widely used in English-speaking countries for about 20 years. However, its meaning has evolved since its first definition as “cognitive and social skills which determine the motivation and ability of individuals to gain access to, understand and use information in ways which promote and maintain good health”, to be most often centered on the narrower meaning of “functional health literacy”, which is the ability to read and understand written medical documents. This narrow definition excludes illiterate populations and don't take into consideration the social skills. Moreover, it doesn't take into consideration the barriers erected by the culture. Working in the field of health communication, we propose the broadest concept of “health culture”, which encompasses health literacy as well as all the representations that influence people decisions about their health. The health culture approach makes it possible to base strategies, messages and communication tools on the representations of the target populations, in order to sensitize them to the behavioral changes promoted by this communication. We show some examples of its application in public health programs in sub-Saharan Africa: breastfeeding, pregnancy support, Ebola virus disease, HIV testing, tuberculosis, and COVID-19.

## Introduction

Voici une vingtaine d'années, des médecins et spécialistes de santé publique américains, britanniques et australiens ont avancé le concept de « *health literacy* » qui est aujourd'hui l'objet de nombreux travaux dans des pays anglophones mais, malheureusement, encore peu connu dans les pays francophones, particulièrement en France et encore davantage dans les pays francophones africains.

Le concept est né du constat « *de l’échec de programmes antérieurs d’éducation à prendre en compte les déterminants sociaux et économiques de la santé […] ce qui a conduit à sous-estimer le rôle potentiel de l’éducation à la santé* », a expliqué le professeur de santé publique australien Nutbeam [8]. « *Des campagnes qui se concentraient uniquement sur la transmission de l'information sans prendre en compte la condition sociale et économique des individus n'atteignaient pas leurs objectifs*. »

**Figure 1:**
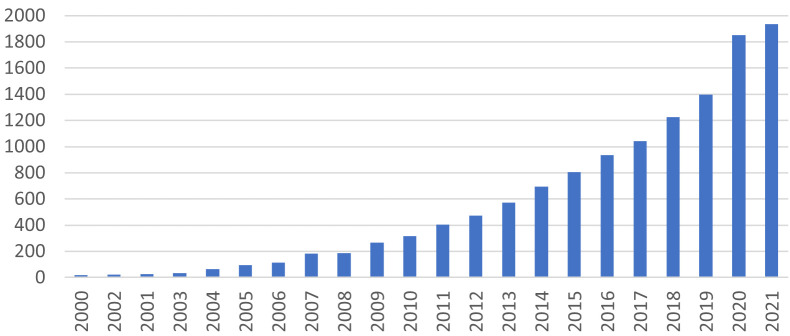
Nombre d'articles comportant « *health literacy* » dans leur titre ou leur résumé Number of articles with “health literacy” in their abstract or main text

La définition généralement reprise de *health literacy* est celle formulée par l'OMS en 2009 : « *Cognitive and social skills which determine the motivation and ability of individuals to gain access to, understand and use information in ways which promote and maintain good health* » [9], que nous pouvons traduire par « *les compétences intellectuelles et sociales qui déterminent la motivation et la possibilité qu'ont les personnes à obtenir, comprendre et utiliser l'information de façon à favoriser et conserver une bonne santé* ». Le nombre d'articles consacrés à la *health literacy* ou la mentionnant a explosé depuis une dizaine d'années (Fig. 1)^1^. Aux États-Unis, les universités de Harvard, de l'Arkansas, du Maryland, Emory, de l'Ohio et du Kentucky, pour ne citer que quelques exemples, ont des départements ou des laboratoires de *health literacy*, tandis que l'Académie nationale des sciences, de l'ingénierie et de la médecine anime un bureau de la *health literacy*. Il en est de même dans des universités ou des instituts du Québec, de Sydney, de Maastricht, de Vienne, de Varsovie, de Dublin, d'Athènes, de Bilthoven (Pays-Bas), de Murcia (Espagne), de Sofia ou de Louvain. Des gouvernements ont mis en place des comités, des bureaux ou des programmes en charge de la *health literacy*, comme au Canada, en Australie, en Grande-Bretagne ou aux États-Unis.

Si des auteurs pouvaient écrire dès 2009 que « *la* health literacy *est devenue un phénomène international* », le phénomène s'arrêtait encore aux portes de la francophonie. Il y a enfin pénétré depuis quelques années, davantage en Suisse et en Belgique qu'en France. Santé Publique France propose aujourd'hui une dizaine de documents sur cette thématique, dont la majorité date de 2017. Un Réseau francophone de « littératie en santé » regroupe une cinquantaine de chercheurs, tous rattachés à des organismes européens. Malgré cela, 110 seulement des 12 906 articles publiés jusqu'en 2022 et contenant « *health literacy* » ou « littératie » dans leur résumé ou leur texte ont au moins un de leurs auteurs affilié à un organisme français^2^.

## Mots et Concepts

Le contenu du concept de *health literacy* a toujours été ambigu. Alors que Nutbeam le considérait comme une façon de « *prendre en compte la condition sociale et économique des individus* » et que la définition de l'OMS mentionne « *les compétences intellectuelles et sociales* », un rapport du Comité sur la *Health literacy* de l'Association médicale américaine publié en 1999 n’évoquait ni la condition sociale, ni la condition économique et se concentrait uniquement sur « *la capacité à lire et comprendre les notices de médicaments, les ordonnances, les compte rendus médicaux et tout le matériel médical nécessaire pour que le patient réussisse son parcours* », ce qu'on appelle la *health literacy* fonctionnelle [2]. « *Cette définition étroite de la* health literacy *laisse de côté une grande partie de la signification et du but de la connaissance* », a écrit Nutbeam, car il existe « *différents “types” de* literacy *et d'utilisation de celle-ci dans la vie quotidienne* » [8]. Pour Rudd *et al.*, cette définition « *encourage une myopie concentrée sur le manque de maîtrise de la lecture et de l’écriture et ignore les barrières érigées par la culture, la langue et les conceptions du personnel de santé* » [13]. « *La* health literacy *ne dépend pas des compétences en écriture et en lecture, comme cela a été démontré dans des pays où l'analphabétisme est élevé* », ont souligné les professeurs d’éducation à la santé Diane Levin-Zamir et Jane Wills [6].

Voici 20 ans, les uns appréhendaient donc l'individu dans son cadre social, les autres dans sa relation avec les documents émanant du corps médical. Les nombreux programmes de *health literacy* lancés depuis relèvent le plus souvent de la seconde interprétation, notamment en France.

Différentes traductions françaises de *health literacy* ont été proposées comme « culture sanitaire » [23], restrictive car elle évoque l'hygiène davantage que la santé, « compétence informationnelle en santé », à la fois complexe et restrictive car il ne s'agit pas seulement d'information, ou encore « compétences en matière de santé », relativement fidèle à l'anglais mais, elle aussi, restrictive [5].

L'expression la plus utilisée est « littératie en santé », que des chercheurs de l'université du Québec définissent comme « l*a capacité d'une personne à comprendre et à utiliser le langage, les chiffres, les images et les technologies afin d’échanger, d'interagir avec les autres, de saisir son environnement, d'acquérir de nouvelles connaissances, de développer son plein potentiel et d’être un citoyen à part entière* » [14], tandis que Santé Publique France écrit : « *par “littératie en santé”, l'on entend le résultat de l'interaction entre les capacités d'une personne à reconnaître son besoin d'information en matière de santé, à trouver cette information, à la comprendre et à l'utiliser pour prendre des décisions éclairées sur sa santé, et les exigences du système de santé* » [1].

La littératie en santé est généralement considérée dans la perspective de l’éducation thérapeutique, comme une intervention destinée à améliorer « *les conditions d'une interaction satisfaisante avec les malades* » [7]. L’éducation thérapeutique fait l'objet de nombreuses études et programmes, dans le cadre d'une démarche qui place le malade au centre de l'organisation des soins, cherchant à transformer la passivité du « patient » en une gestion active de sa santé. La littératie a trouvé sa place comme cadre théorique de ces études, mais le concept de *health literacy*, forgé en réaction aux échecs de campagnes de santé publique, va au-delà de la relation soignants-patients de l’éducation thérapeutique.

L'acception étroite du concept ne peut servir de cadre pour comprendre les déterminants des comportements en santé publique et concevoir des stratégies de communication pour influencer ces derniers. Nous travaillons donc avec l'acception élargie de *health literacy* proposée en 2012 par Rudd, McCray et Nutbeam : « *la conception étendue de la* health literacy *incluant les actions sociales, politiques et individuelles, il faut prendre en compte à la fois les compétences des individus et des communautés et les caractéristiques des institutions et des professionnels qui peuvent renforcer ou inhiber les actions des individus ou des communautés* » [13]. Nous proposons de traduire *health literacy* par « culture en santé » qui recouvre l'ensemble des connaissances et representations d'un individu en matière de santé, sa compréhension d'une question de santé, bien sûr, mais aussi ses peurs, ses espoirs, sa perception des autorités de santé et des interventions médicales et non médicales. Après la dichotomie qu'a connue le concept de *health literacy*, peut-être serait-il d'ailleurs utile aujourd'hui pour les tenants anglophones de son acception large de promouvoir l’étude d'une *health culture*, incluant la *health literacy*.

La culture en santé fournit le cadre d'une approche de la communication basée sur les représentations de la population. Tournant le dos aux démarches verticales, elle oriente vers des stratégies de communication basées sur les faits, si nous pouvons nous permettre un détournement de l'expression « *Evidence-Based Medicine* », les faits étant ici la culture en santé de la population sur la question de santé concernée.

Nous proposons succinctement quelques exemples de la mise en oeuvre de cette approche dans des programmes que nous avons menés en Afrique de l'Ouest. Des enquêtes anthropologiques nous ont fourni des photographies de la culture en santé sur la base desquelles nous avons proposé des stratégies et outils de communication.

## Grossesse et Allaitement Maternel

Nous étions chargés, en 2013, d’élaborer une stratégie et des outils de sensibilisation pour les volontaires de deux programmes en République démocratique du Congo (RDC), l'un destiné aux femmes enceintes, l'autre aux jeunes mères. Le travail d'une anthropologue, qui a conduit des focus groupes, nous a fourni des éléments sur les représentations des femmes et de leur entourage, maris, belles-soeurs et bellesmères^3^.

L'une des principales recommandations à promouvoir auprès des femmes enceintes était de se rendre en consultation prénatale dès le début de la grossesse, or nous avons découvert que ce « début » n’était pas toujours bien défini, les femmes estimant souvent n’être enceintes que lorsqu'elles sentaient les mouvements du foetus. Ceci nous a amenés à inclure dans les documents d'aide de visite des dessins montrant que le foetus existe dès l'aménorrhée. Nous avons, de même, identifié une méconnaissance des risques spécifiques liés au paludisme chez la femme enceinte et du fait que la maladie est transmise par des moustiques nocturnes, ce qui limitait la portée de la recommandation de dormir sous une moustiquaire, à quoi s'ajoutait que l'odeur des moustiquaires, due à l'insecticide qui les imprègne, était considérée comme nocive. Nous avons fourni dans les outils de communication les explications nécessaires.

L'enquête sur les perceptions de l'allaitement exclusif jusqu’à six mois nous a apporté des éléments qui expliquaient pourquoi les données de santé en RDC indiquaient que la grande majorité des mères connaissait cette recommandation, mais qu'une minorité seulement l'appliquait. Des femmes disaient que cette recommandation était très bien… mais qu'elles connaissaient leur bébé et que celui-ci avait besoin d'eau et de bouillie dès les premières semaines. Les principales raisons étaient qu'en Afrique un bébé a besoin de boire de l'eau comme un adulte car il fait chaud, que le lait ne contient pas toute la nourriture nécessaire, que quand on est mal nourrie on ne peut pas avoir un lait suffisamment nutritif, enfin qu'elles ne produisaient pas assez de lait.

Notre réponse a été de ne pas utiliser d'injonctions ni dénoncer ce qui pourrait être mauvais pour un bébé, les femmes pensant savoir mieux que quiconque ce qui est bon pour leur enfant. Nous avons cherché à apporter aux femmes et à leur entourage des informations susceptibles d'encourager l'allaitement exclusif, par exemple en expliquant que le lait contient toute l'eau dont un bébé a besoin et tous les nutriments de l'alimentation d'un adulte, que le lait d'une femme dénutrie est quand même nutritif et qu'un bébé à qui on donne de l'eau ou de la bouillie tête moins, ce qui entraîne une baisse de la lactation.

## Maladie a Virus Ébola

Nous avons été sollicités par le ministère de la Santé togolais, en 2015, pour proposer une stratégie et des outils de communication pour la préparation à l'arrivée éventuelle dans le pays de la maladie à virus Ébola qui touchait alors trois pays de la sous-région.

Une enquête anthropologique a montré que les messages diffusés par le ministère de la Santé avaient très bien atteint la population, le numéro vert étant très largement connu, et que le message le plus retenu était qu'il fallait « éviter la viande de brousse », alors que la transmission épidémique est exclusivement interhumaine, et qu'il fallait se laver les mains et éviter les accolades et les serrements de main [16]. Une idée répandue était qu’Ébola était un prétexte pour réinstaurer les mesures de protection de la faune sauvage appliquées par l'ancien dictateur Eyadema Gnassingbé. L'enquête a également mis en relief que si les mesures de distanciation et le lavage des mains avaient été respectés au début de la campagne de sensibilisation, ceci était de moins en moins le cas au fil du temps.

Nous avons proposé une réorientation de la communication abandonnant la mention des animaux, informant sur les voies de transmission de la maladie (le contact avec les malades et les morts, et leurs fluides corporels), mais ne recommandant pas d'appliquer des gestes de prévention tant que la maladie n’était pas présente dans le pays. La maladie à virus Ébola ayant heureusement épargné le Togo, cette stratégie et les outils de communication vidéo et imprimés produits pour sa mise en oeuvre n'ont pas eu l'occasion d’être déployés.

## Promotion du Test de Dépistage du VIH

L'Onusida et le Fonds mondial contre le sida, le paludisme et la tuberculose (FM) nous ont demandé, en 2018, de proposer une stratégie de communication pour augmenter la demande de tests de dépistage du VIH, ce qui nous a amenés à travailler sur cette question en Côte d'Ivoire, avec le Programme national de lutte contre le sida.

Une enquête anthropologique qualitative a mis en évidence que l'existence et la fonction des tests de dépistage étaient largement connues et que la perception des médicaments antirétroviraux contre le VIH était contradictoire : une majorité des enquêtés les connaissait et disait qu'ils permettent de vivre en bonne santé, mais le VIH était toujours fréquemment associé à la mort [22]. De ce fait, l'annonce d'un diagnostic positif au VIH était souvent considérée davantage comme une condamnation à mort que comme la possibilité d'accéder à des traitements qui repousseraient cette mort. Nous avons préconisé une reprise de la communication de masse pour changer l'image de l'infection à VIH, communication abandonnée depuis une quinzaine d'années au profit de communications ciblées, et de séparer nettement « VIH » et « sida », notamment en bannissant l'expression « VIH/sida » car le premier n'entraîne pas nécessairement le second.

Nous avons été intrigués par le fait que, selon notre enquête, les jeunes pensaient davantage que les anciens que le VIH équivaut à la mort, alors que le sida est au programme de plusieurs années des classes primaires et secondaires. Nous avons étudié les livres scolaires et découvert que la grande majorité d'entre eux présentaient l’évolution vers la mort des personnes infectées par le VIH comme inéluctable, mentionnant parfois les tests mais quasiment jamais les traitements [15]. Ces idées fausses semblaient être compensées chez les adultes par leur expérience personnelle, le fait qu'ils connaissaient des personnes infectées sous traitement ou en avaient entendu parler.

## Promotion du DÉPistage de la Tuberculose

Le FM et le Programme national de lutte contre la tuberculose de Côte d'Ivoire nous ont demandé, en 2018, de formuler des propositions de communication pour améliorer le dépistage de la tuberculose, dont l'OMS estime que 50% des cas ne sont pas diagnostiqués en Afrique, 45% en Côte d'Ivoire [10, 11].

Une enquête préliminaire menée auprès d'une cinquantaine de soignants et membres d'ONG intervenant sur la tuberculose nous avait laissé penser que les principales explications du sous-diagnostic de la maladie, en plus de difficultés sociales et des insuffisances de l'offre de soins, étaient une méconnaissance de la tuberculose et une confiance dans la médecine traditionnelle censée détourner les malades tuberculeux des structures sanitaires. Or, une enquête socio-anthropologique quantitative nous a montré que la population avait une très bonne connaissance de l'existence et de la gravité de la tuberculose, que 28,25% seulement pensaient que les médicaments traditionnels sont efficaces contre la tuberculose, tandis qu’à la question « À quel type de médecine recourez-vous lorsqu'il s'agit de la tuberculose ? » 10,25% avaient répondu « la médecine traditionnelle » [19]. En revanche, la moitié de la population seulement savait que le traitement de la tuberculose est gratuit. En outre, la tuberculose était très majoritairement associée au sida, ce qui engendrait une peur de la discrimination. Nos résultats ont ainsi brossé une « culture » de la tuberculose inattendue, sur la base de laquelle nous avons formulé des recommandations de communication. D'abord, contrer l'influence de la médecine traditionnelle ne devait pas être un axe de la sensibilisation au dépistage de la tuberculose, tandis qu'il fallait populariser la gratuité des traitements. Ensuite, il fallait dissocier VIH et tuberculose, souvent associés dans la communication sur le VIH.

## Covid-19

L'Organisation Ouest Africaine de la Santé (OOAS), agence santé de la Communauté économique des États de l'Afrique de l'Ouest (CEDEAO), nous a demandé en 2020 de mener une enquête socio-anthropologique dans 5 de ses États membres, pour étudier les représentations sur le Covid-19 et baser une stratégie et des outils de communication sur ces représentations [20, 21].

Les volets qualitatif et quantitatif de l’étude ont mis en évidence un sentiment très largement répandu de non-exposition au Covid-19 : croyance que si le Covid-19 existe sur d'autres continents il n'est pas présent dans le pays, ignorance ou sous-estimation des facteurs de risque que sont l’âge et l'obésité, ignorance de la transmission asymptomatique. Les théories complotistes et idées fausses qui circulent sur les réseaux sociaux ne semblaient, en revanche, pas jouer un rôle mesurable dans le non-respect des gestes barrières et le manque d'adhésion à la vaccination. Notre recommandation a été d’éviter les injonctions de respecter les gestes barrières et de se faire vacciner, pour concentrer les efforts de communication sur la transmission des informations et explications nécessaires sur la réalité de la pandémie dans les pays africains, les facteurs de risque et les voies de transmission.

## Discussion

Les exemples de manque d'adhésion de la population à des mesures de santé publique sont nombreux, souvent lors de campagnes de vaccination mais aussi par exemple, spectaculairement, lors d’épidémies de maladie à virus Ébola. Des communications maladroites ont souvent contribué à nourrir les doutes, doutes qui se transforment en méfiance, puis en hostilité quand, au lieu de comprendre les raisons, fondées ou infondées, de ces doutes et d'y répondre, les responsables de la santé publique se contentent de marteler les mêmes messages [4, 18]. La prise en compte de la culture en santé de la population est indispensable pour concevoir une communication adaptée, limiter les risques d'incompréhension et de méfiance, faciliter l'adhésion [3]. Cette culture en santé peut être évaluée par des études de sciences sociales, études qui doivent inclure les connaissances et représentations sur la question de santé concernée, mais également sur le système de santé et les promoteurs des mesures de santé publique.

On n'a jamais autant consacré d'articles, de rapports, de réunions et de webinaires à la circulation d'idées fausses sur une question de santé que depuis le début de la pandémie de Covid-19, plus précisément depuis que le Directeur général de l'OMS, Tedros Adhanom Ghebreyesus, a déclaré le 15 février 2020 : « *Nous ne combattons pas seulement une épidémie, nous luttons aussi contre une infodémie. Les informations fausses se propagent plus vite et plus facilement que ce virus, et elles sont tout aussi dangereuses* ». Que les informations fausses se propagent aujourd'hui rapidement sur internet est incontestable, mais c'est aussi le cas des informations exactes qui s'affichent désormais sur les smartphones de personnes autrefois coupées de toute information médicale. Un des enjeux de santé publique, celui de la communication sur le Covid-19, est de mesurer leur dangerosité réelle en étudiant leur impact sur la culture en santé, la culture du Covid-19 [17].

La communication en santé a pour but un changement de comportement : port du préservatif, lavage des mains, utilisation d'une moustiquaire, hygiène de vie, gestes barrières, vaccination… Ces comportements ne peuvent être durablement obtenus par l'injonction, l'inspiration de la crainte ou la culpabilisation. Des auteurs se demandaient déjà en 2007 : « *Pourquoi les plans de communication en santé se ressemblentils tellement, alors que les populations, les problèmes, la culture et le vécu concret sont si différents ?* » [12]. Pour être efficace, la communication en santé doit se fonder sur la culture en santé de la population pour déterminer une stratégie et concevoir des messages et des outils de communication qui gagneront l'adhésion aux changements de comportement désirés. L'impact de ces messages sera ensuite mesuré et ils seront modifiés si les représentations de la population évoluent. Une démarche de culture en santé est ainsi une démarche circulaire, prenant sa source dans la population, passant par les spécialistes, puis revenant à la population, par opposition à la démarche verticale de spécialistes qui conçoivent en conclave les messages qu'ils estiment les meilleurs. Peut-être n'est-elle, finalement, que l'extension au niveau de la communication de l'approche que bien des personnels soignants, des services de santé ou des ONG suivent quotidiennement auprès de leurs patients ou des communautés dans lesquelles ils interviennent.

Élever la culture en santé de la population, c'est aussi lui donner les moyens de sa responsabilisation [8], promouvoir une communication en santé de type démocratique, une communication qui ne soit pas fondée sur des injonctions d'en haut, des intimidations, des discours martiaux culpabilisants, mais qui sollicite l'esprit civique, la compassion, le sens des responsabilités et la fraternité [24].

## Liens D'intérêts

L'auteur est consultant en communication santé. Les travaux mentionnés ont été financés par l'Onusida, le Fonds mondial, COOPI, l'Agence Française de Développement, SIS International et le gouvernement de la République togolaise.
